# Oral White Lesions Associated with Chewing Khat

**DOI:** 10.1186/1617-9625-2-3-145

**Published:** 2004-09-15

**Authors:** Meir Gorsky, Joel B Epstein, Harel Levi, Noam Yarom

**Affiliations:** 1 Department of Oral Pathology and Oral Medicine, The Maurice and Gabriella Goldschleger School of Dental Medicine, Tel Aviv University, Tel Aviv, Israel; 2 Medical-Dental Staff, British Columbia Cancer Agency, Vancouver, B.C., Canada; 3 The Maurice and Gabriella Goldschleger School of Dental Medicine, Tel Aviv University, Tel Aviv, Israel; 4 Department of Oral Medicine and Diagnostic Sciences, 801 South Paulina St., Chicago, IL 60612, USA

## Abstract

**Introduction:**

Khat is a cultivated plant whose leaves when chewed elevate mood. Unlike the chewing of betel nut, no association between the white oral mucosal lesions in khat users and oral malignancies has been reported. Chewing of khat has been documented in many countries and has increased with worldwide migration. The impact of chewing khat upon the oral mucosa is essentially unknown.

**Purpose:**

The purpose of this study was to assess the occurrence of oral white changes in chronic khat chewers. Oral mucosal changes in a group of 47 Yemenite Israeli men over 30 years of age, who had chewed khat more than 3 years, were compared to those of 55 Yemenite men who did not chew.

**Results:**

White lesions were significantly more prevalent in the khat chewers (83%) compared to the non chewing individuals (16%) (P < 0.001). White oral lesions were identified primarily on the lower buccal attached gingival mucosa, the alveolar mucosa and the lower mucobuccal fold on the chewing side (p < 0.001). There was no significant association between the occurrence of the white lesions and smoking. Even though the majority of the white lesions (85.4%) were homogenous, 71.4% of the non homogenous lesions were identified in khat chewers. Vital staining with toluidine blue and exfoliative cytology was conducted on a subset of patients with homogenous and non-homogenous oral lesions, and there were no findings suspicious for pre-malignant or malignant changes.

**Discussion:**

This study demonstrated a relationship between khat chewing and oral white lesions, which we attribute to chronic local mechanical and chemical irritation of the mucosa. Our findings also suggest that mucosal changes associated with khat are benign, however, this initial study requires further studies including follow-up of khat users to confirm the current findings, including the likely benign changes associated with chronic use and histologic findings of clinical lesions.

## Introduction

**Figure 1:**
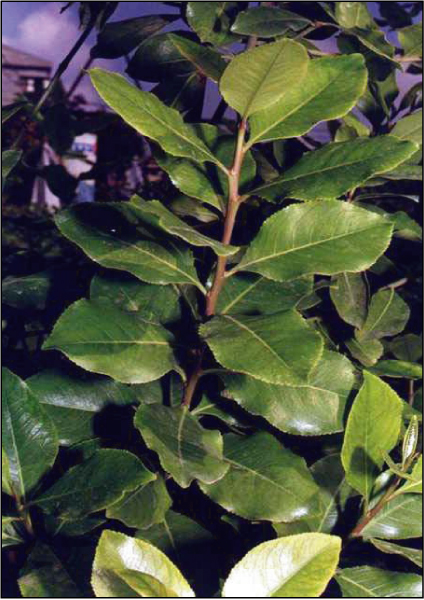
**Khat leaves**.

Catha edulis, also known as khat or qat, is a cultivated plant (Figure 1) whose chewed leaves cause effects upon mood. It was first described in 1237 [1]. Khat is a green bush that is found mainly in East African areas as well as in Saudi Arabia and Yemen [2,3]. The medical properties vary based on the color of its leaves and red leaves have stronger medical properties than the green leaves [4]. Cathinone, found in khat leaves, is probably the alkaloid that has stimulating effects upon the central nervous system resulting in mood elevation and euphoria [3,5].

**Figure 2:**
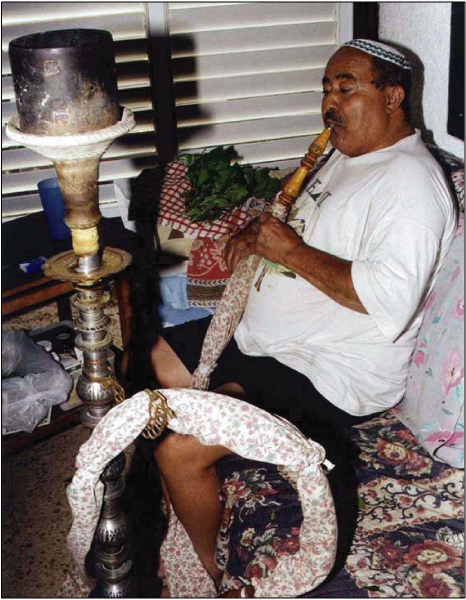
**Chewing khat and smoking of the nargila**.

Khat leaves, which are generally placed in the mouth in the lower distal mucobuccal fold, are usually chewed during social-cultural meetings where the chewing process may take up to 5 hours [2]. Since the process of khat chewing has a drying effect on the oral mucosa, its users tend to consume a great quantity of fluids [6]. Some of the khat users also supplement their chewing practice with smoking of the Nargila pipe (water pipe) simultaneously [4] (Figure 2). Side effects that are believed to be related to the chewing of khat include elevation of blood pressure, tachycardia, hyperthermia, increased sweating, muscular weakness, loss of appetite, spermatorrhea and some gastrointestinal disturbances [7-10].

The practice of chewing khat is comparable to the process of betel nut chewing in eastern countries like India, Bangladesh, Taiwan, Malayasia and the Philippines. Betel nut, also known as areca nut, is usually chewed in the form of a betel quid that is a mixture of betel nut leaves wrapping, lime paste, some flavoring agents, and other ingredients that vary with demography. The mixture, which frequently contains tobacco, has pharmacologically and psychologically stimulating properties [11]. Although oral carcinogenicity is attributed primarily to the tobacco component of the betel quid, the mixture, even without tobacco, has carcinogenic potential [12], and is classified by the International Agency for Research on Cancer  as a class 1 carcinogen.

Only a limited number of studies assess the potential impact of khat use upon oral health. It has been speculated that there might be an association between khat chewing and oral malignancies [13]. Macigo and colleagues [14] reported that although they found oral leukoplakia in few of the khat users examined in Kenya, and no significant association between chewing khat and leukoplakia was seen. Hill and Gibson [2] reported that oral white lesions, resembling those of frictional keratosis, were noted in 50% of khat chewers in Yemen. Since none of the lesions was suspected for malignancy, no biopsies were performed.

The cultural use of communal khat chewing is common among the Yemenite Jews in Israel ranging from twice a week to daily usage. Although two articles (published in Hebrew) described the effect of khat chewing among the Yemenite Jews in Israel, no evaluation of the possible effect on the oral mucosa was reported [15,16]. In a search of the English literature we did not find any other publications assessing oral lesions in khat chewers other than those reviewed above. The purpose of the present study was to assess the prevalence and the clinical findings of oral white lesions in a chronic khat chewing population.

## Materials and methods

A population survey was performed on 1500 Yemenite Israeli Jews of one city in Israel known to have a large population of Yemenite Jews. Only individuals who provided a history of chewing khat for more than 3 years, who were older than 30 and both of whose parents were born in Yemen, were included. The subjects underwent a personal interview that included questions regarding their smoking habits, khat chewing habits, and the duration of their habits (if positive). A group of 102 individuals who met the requirements were identified, and clinical examinations were performed under standard clinical conditions. In this study, oral white lesions in users of khat (khat-induced leukoplakia) were defined as a white lesion at the site of khat use that cannot be removed and is not representative of any other white lesion [16,17].

The study group included 47 Yemenite Jewish Israeli males. A group of 55 men randomly selected among the 1500 surveyed men, who had never used khat, matched for age and ethnic origin (both parents born in Yemen), served as the control. Each group was divided into two subgroups: tobacco users and non-users.

Clinical examination for oral mucosal white lesions that could not be scraped off was performed by two of the authors (M.G. and H.L.), using standard dental office lighting. The examiners were calibrated by first examining a group of 25 khat chewers with oral white lesions and complete agreement was seen.

The data were analyzed by means of the Chi-square and Variance tests of significance. P ≤ 0.05 was considered statistically significant.

## Results

Thirty-two (68%) of the 47 khat chewers were also cigarette smokers and 25 (45.5%) of the non-chewers smoked cigarettes (Table 1). There was no difference in the mean chewing duration between the 32 smokers (23.8 years) and the 15 non-smokers (24.1 years) (p = 0.93). No difference was found in the mean frequency of khat chewing each week, 3.5 times per week in the smoking and chewing subjects and 3.33 in the non-smoking chewers (p = 0.76). The mean duration of each chewing session in the smokers was 4.12 hours compared to 3.5 hours in the non-smoking chewing group (p = 0.066). Khat chewers who also smoked consumed significantly more cigarettes compared to the non-chewers who smoked tobacco (mean 29.5 per day compared to 22.3 cigarettes per day respectively p = 0.03).

**Table 1:** The characteristics of 102 khat chewers and non-chewers

	**Khat chewers**		**Non-chewers**	
		
	**No.**	**Age (range)**	**No.**	**Age (range)**
Tobacco users	32	50.6 (35–84)	25	40.2 (30–53)
Non-smokers	15	58.6 (44–74)	30	50.6 (37–67)
Total	47	53.2 (35–84)	55	45.9 (30–67)

**Figure 3:**
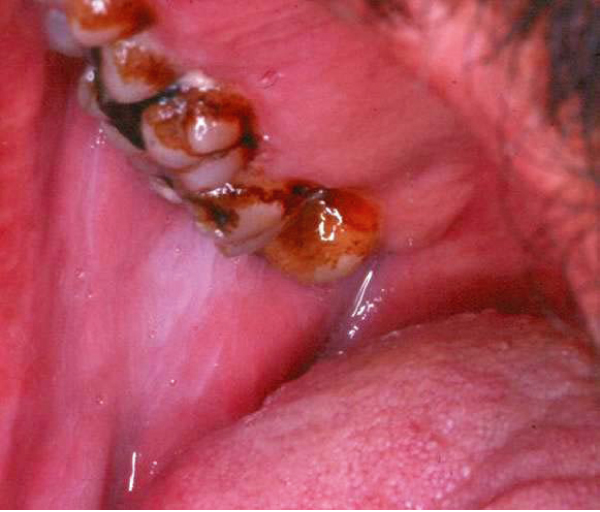
**White lesion of the buccal mucosa in a 55-year-old individual who is a non smoker and who chewed khat for over 25 years**.

White lesions on the oral mucosa (Figure 3) were most common on the lower buccal attached gingival mucosa, the alveolar mucosa and the lower mucobuccal fold at the second premolar and molar areas. White lesions were identified in 39 subjects (83%) of the khat chewers compared to only 9 individuals (16.3%) of the control group (p < 0.001) (Table 2). White lesions were identified in 48 individuals, and in 41 (85.4%) persons they were completely homogenous. Five of the seven non-homogenous lesions (71.4%) were in khat chewers. These findings indicate an approximately threefold higher risk of developing non-homogenous white changes in khat chewers compared to non-chewers. A significantly higher occurrence of white lesions was seen on the chewing side (37 subjects (100%) versus 3 lesions (7.7%) on the non-chewing side (p < 0.001). Although 3 patients (8.1%), who were also smokers, had white lesions on the non-chewing side, white lesions were noted at the chewing site of all chewers. Two patients chewed on both sides, and white lesions were identified in those patients in both sides of their oral cavity.

**Table 2:** The prevalence of white lesions on the oral mucosa of the 102 individuals

	**Khat chewers**		**Non-chewers**	
		
	**Smokers (%)**	**Non-smokers (%)**	**Smokers (%)**	**Non-smokers (%)**
Individuals with white lesions	27/32 (84.4)	12/15 (80.0)	5/25 (20.0)	4/30 (13.3)*
Individuals with no white lesions	5/32 (15.6)	3/15 (20.0)	20/25 (80.0)	26/30 (86.7)

*All were diagnosed clinically as frictional keratosis

No white lesion was felt to be clinically suspicious for malignant or premalignant changes and no one agreed to undergo biopsy of his white lesion. Every fourth individual with mucosal changes underwent further clinical evaluations. Vital tissue staining with toluidine blue and exfoliative cytology tests were performed on a subset of 7 homogenous and 3 non-homogenous khat chewers' white lesions. Since no clinical toluidine blue dye uptake was noted and no atypical epithelial cells were observed, no suggestive premalignant or malignant changes were identified in the subset of patients.

## Discussion

The subjects of the study and the control group were matched for age and shared the same ethnic background. Since the Yemenite Jews, who are part of the Eastern origin Jews, are dark skinned, oral pigmentation was noted in about two thirds of the individuals [19]. Leukoedema is common in smokers who also have a pigmented oral mucosa [20] and it may be expected that leukoedema-like changes would be seen more commonly in cigarette users. However, the prevalence of white lesions in khat chewers who were non-smokers (80%) was significantly higher than in the smoking control group (20%) (p < 0.05), indicating a possible association between khat chewing and white oral mucosal lesions. The findings of a significant association between the site of the white changes (p < 0.001) and the chewing side further supports a cause and effect relationship.

Interestingly, khat chewers who smoked consumed significantly more cigarettes daily (p = 0.03). It would be expected to find more white oral lesions in the smoking and chewing subjects. However, the non-significant difference in the prevalence of the white changes between the smoking and chewing group (84%) and the non-smoking chewing group (80%) (p > 0.05) further supports a direct association between oral white mucosal plaques and khat chewing.

In a study that evaluated the mucosal pigmentation on the khat chewing site (unpublished data) we speculated that the increase in the prevalence and the intensity of the brownish-gray color on the chewing site is directly related to chemical components in the khat leaves. We believe that such chemicals may also contribute to the development of oral white changes. In all khat chewers, white lesions were noted on the chewing side, demonstrating a probable association between the local mechanical and possible chemical irritation of the mucosa and the white mucosal lesions. Mechanical irritation was also proposed by Hill and Gibson in their study of khat chewers in north Yemen [2]. It is likely that white changes are related to the combination of mucosal dryness, as reported by khat users [6], and exposure to chemical and mechanical irritation.

Chewing of betel nut is a more common habit worldwide than khat chewing. Unlike khat chewing, in selected populations women may be more common users of betel nut than men [21]. We did not identify any women who used khat among the Yemenite residents in Israel.

The International Agency for Research on Cancer (IARC) reported that there is sufficient evidence that chewing betel quid containing tobacco is a class 1 carcinogen [22]. Betel nut (areca nut) is implicated in oral epithelial lesions including oral leukoplakia and sub-mucous fibrosis, both of which represent premalignant conditions [23]. The increased risk of cancer in users of betel has been documented [24-26] and it has been suggested that there was sufficient evidence that chewing betel quid without tobacco is carcinogenic in humans [22]. The betel chewers' mucosa (BCM) is characterized by a localized brownish-red discoloration of an irregular and rough oral mucosa that may be found together with other oral mucosal lesions such as leukoedema and leukoplakia [27]. Lichenoid oral lesions at the chewing site have also been reported among betel quid users [28]. A study from Cambodia reported that 61% of betel quid users had mucosal changes that were directly associated to their chewing habit and only 3% (all smokers) had white homogenous lesions occurring in the chewing area of the mucosa [29]. Similar to our speculation on the cause of leukoplakia in khat chewers, it is believed that the BCM is either a result of direct chemical action of the components in the mixture or due to the traumatic effect of the chewing, or both [30].

Although an association between khat chewing and oral malignancy was speculated [13] it has not been proven. Kennedy and colleagues, [4] who examined the oral cavity of 706 khat users in North Yemen, reported that no oral malignancies were found. Although based on the clinical appearance (no red component and no ulcerations), no white lesion in the present study was clinically suspicious for malignancy or premalignancy, some clinical tests were performed. Vital staining with toluidine blue (tolonium chloride) has been shown in a multicenter controlled clinical trial to have a sensitivity of 96.7% in identification of lesions that upon biopsy represented SCC or CIS, and had a positive predictive value of 32.6% [31], in addition, lesions with severe dysplasia and chromosomal changes associated with SCC are detected by toulidine blue [32]. Cytologic examinations using the OralCDx brush biopsy (OralCDx^®^) were performed on a sample of ten lesions, including three non-homogenous white lesions, and no suspicious lesion or cells that have atypical appearance have been found. OralCDx has been reported to provide 100% sensitivity [33]. A recent study of 243 OralCDx specimens reported a false positive rate of approximately 60%, and a positive predictive value of 38% [34], another report showed sensitivity of 61.5% and a specificity of 94.3% [35]. Thus tolonium chloride and OralCDx represent adjuncts for identification and diagnosis of oral premalignant and malignant disease that are unlikely to provide false negative results. The findings in the current trial suggest benign mucosal changes due to khat chewing.

Increasing use of khat is occurring worldwide with worldwide immigration from areas of high use. Recent reports of increasing use of khat in the United States, and classification of khat by the Drug Enforcement Administration as a schedule one drug (News-week, Sept 30, 2002, p. 35), have drawn attention to the use of khat, and due to this, it is likely that oral lesions as reported in this trial will be seen throughout the world. Since the information available on the use of khat and oral health in general and the nature of the white mucosal changes in particular is very limited, further studies including longitudinal clinical inspections and biopsies of the oral tissues changes of khat chewers are needed.

## Competing interests

The authors declare that they have no competing interests.
